# Invasive‐dominated grasslands in Hawaiʻi are resilient to disturbance

**DOI:** 10.1002/ece3.10948

**Published:** 2024-03-19

**Authors:** Stephanie Yelenik, Eli Rose, Susan Cordell

**Affiliations:** ^1^ U.S. Geological Survey Pacific Island Ecosystem Research Station Hawaiʻi National Park Hawaii USA; ^2^ U.S. Forest Service Institute of Pacific Islands Forestry Hilo Hawaii USA; ^3^ Present address: USDA Forest Service, Rocky Mountain Research Station Reno Nevada USA

**Keywords:** C4 grasses, colonization, community assembly, disturbance, Hawai‘i, invasions biology, invasive grasslands

## Abstract

Non‐native‐dominated landscapes may arise from invasion by competitive plant species, disturbance and invasion of early‐colonizing species, or some combination of these. Without knowing site history, however, it is difficult to predict how native or non‐native communities will reassemble after disturbance events. Given increasing disturbance levels across anthropogenically impacted landscapes, predictive understanding of these patterns is important. We asked how disturbance affected community assembly in six invaded habitat types common in dryland, grazed landscapes on Island of Hawai‘i. We mechanically disturbed 100 m^2^ plots in six vegetation types dominated by one of four invasive perennial grasses (*Cenchrus ciliaris*, *Cenchrus clandestinus*, *Cenchrus setaceus*, or *Melinis repens*), a native shrub (*Dodonaea viscosa*), or a native perennial bunchgrass (*Eragrostis atropioides*). We censused vegetation before disturbance and monitored woody plant colonization and herbaceous cover for 21 months following the disturbance, categorizing species as competitors, colonizers, or a combination, based on recovery patterns. In addition, we planted individuals of the native shrub and bunchgrass and monitored survival to overcome dispersal limitation of native species when exploring these patterns. We found that the dominant vegetation types showed variation in post‐disturbance syndrome, and that the variation in colonizer versus competitor syndrome occurred both between species, but also within species among different vegetation types. Although there were flushes of native shrub seedlings, these did not survive to 21 months within invaded habitats, probably due to regrowth by competitive invasive grasses. Similarly, survival of planted native individuals was related to the rate of regrowth by dominant species. Regardless of colonization/competitor syndrome, however, all dominant vegetation types were relatively resilient to change. Our results highlight that the altered post‐agricultural, invaded grassland landscapes in Hawaiʻi are stable states. More generally, they point to the importance of resident communities and their effects on species interactions and seed availability in shaping plant community response to disturbance.

## Graphical abstract

We ask whether most species in disturbed habitats fall into similar categories with respect to being strong competitors versus colonizers using a conceptual framework based on how species assemble into plots over time. Our study uses a replicated disturbance experiment, coupled with close tracking of plant cover and native seedlings, as well as a native outplanting experiment, across six dominant vegetation types, including native and invader‐dominated, in old pasture habitats in Hawaiʻi. Our results highlight that the altered states across formerly grazed landscapes in Hawaiʻi are stable to disturbance, and point to the importance of resident communities and their effects on species interactions and seed availability in shaping plant community response to disturbance.

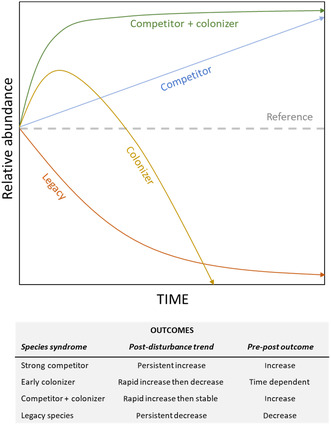

## 1 INTRODUCTION

Land‐use history, disturbance, spatial heterogeneity, and invasive species often interact to alter plant community assembly in anthropogenic landscapes (Denslow, 1980; Morris et al., 2011; Shea & Chesson, 2002; Sousa, 1984). While knowing the mechanisms that lead to invasion can help determine best management practices, this can prove challenging in long‐invaded sites. Disturbances such as land clearing or fire can facilitate invasion by non‐native species with high colonization potential by making resources such as light or water temporarily more available (Davis et al., 2000; MacDougall & Turkington, 2005; Pacala & Rees, 1998; Shea & Chesson, 2002). Such colonizing species may have high seed dispersal rates, be able to quickly exploit resources, or have a combination of these traits (Bolker & Pacala, 1999; Cadotte, 2007). On the other hand, it is possible that dominant species in such invaded areas are those that are best able to competitively draw down resources and maintain positive population growth rates over time, thus driving ecosystem change (HilleRisLambers et al., 2010; MacDougall & Turkington, 2005; Tilman, 1985). Ecosystems are more likely to be dominated by such highly competitive species as time as disturbance increases, as put forth by the intermediate disturbance hypothesis (Sousa, 1984).

Because most landscapes are a mosaic of land‐use history and disturbance, it is possible for both colonizer and competitor species to coexist over broad spatial scales (Cadotte, 2007; Denslow, 1980; Mordecai et al., 2016). Histories of planting invasive species for agriculture and forestry can vary across landscapes, depending on land use (Bennett et al., 2006), and disturbances such as fire are layered over this mosaic in a variety of sizes and return intervals (Fuhlendorf et al., 2006; Warren et al., 2007). Finally, there are underlying patterns of environmental variation such as soil type or climate. All of these factors affect species assemblages in ways that create complex patterns across a landscape and affect future species dynamics (Denslow, 1980; Fuhlendorf et al., 2006). For example, new invasive species may not become dominant if the priority effects of resident species are strong, but these same newly arrived species may be able to occupy and become dominant given certain types of disturbance (Kellner et al., 2011).

Therefore, how future disturbance will affect plant communities in already degraded landscapes is unpredictable (Moles et al., 2007). While a landscape dominated by disturbance‐following colonizer species would be expected to return to a similar species assemblage, a landscape dominated by highly competitive species may shift to disturbance‐followers, at least immediately following disturbance. These scenarios are further complicated by a general lack of understanding about why persistent native species can coexist with invasives in highly disturbed habitats. Native species that persist are often those with early successional or disturbance‐adapted traits such as high seed dispersal rates (Davis et al., 2000; Shono et al., 2006); however, it is also possible that they competitively exclude invasive species or maintain populations in refugia from disturbance (Going et al., 2009; Krawchuk et al., 2020).

Closely tracking community assembly in the few years after disturbance gives insight into the mechanisms that led to current plant communities (Turner, 2010) and elucidates how native species will interact with resident invasive species under continued disturbance. For example, if native species germinate but do not survive over time, this suggests that competition with invasive species rather than restricted colonization is driving native species extirpation. On the other hand, if no seedlings emerge, it is likely that native species are colonization‐limited, perhaps due to a lack of seed dispersal (Seabloom et al., 2003; Yelenik & Levine, 2010). These mechanisms may shift in relative importance along environmental gradients or as resident species shift, altering disturbance outcomes (Moles et al., 2007).

On the island of Hawai‘i, steep environmental gradients and substrate age variation interact to alter species dominance across the landscape (Mueller‐Dombois et al., 2013; Vitousek, 2004). A history of anthropogenic disturbance, and grazing in particular, further increases vegetation heterogeneity (Leopold & Hess, 2017; Perroy et al., 2016). Ranching and other agricultural operations have downsized over time, leaving large areas of old pasture that some organizations are starting to restore or repurpose (Trauernicht et al., 2015; Warren et al., 2019; Yelenik, Rose, & Paxton, 2022). We were interested in how disturbance affected plant community assembly across different dominant vegetation types in areas that have already been altered by grazing and plant invasion, but maintain some native shrub and grassland assemblages. Such areas have proven difficult to restore (Cabin et al., 2002; Questad et al., 2014), and have become prone to greater disturbance over time, in particular, due to development, grazing, military training, and fire (D'Antonio & Vitousek, 1992; Questad et al., 2014; Trauernicht et al., 2015).

**FIGURE 1:**
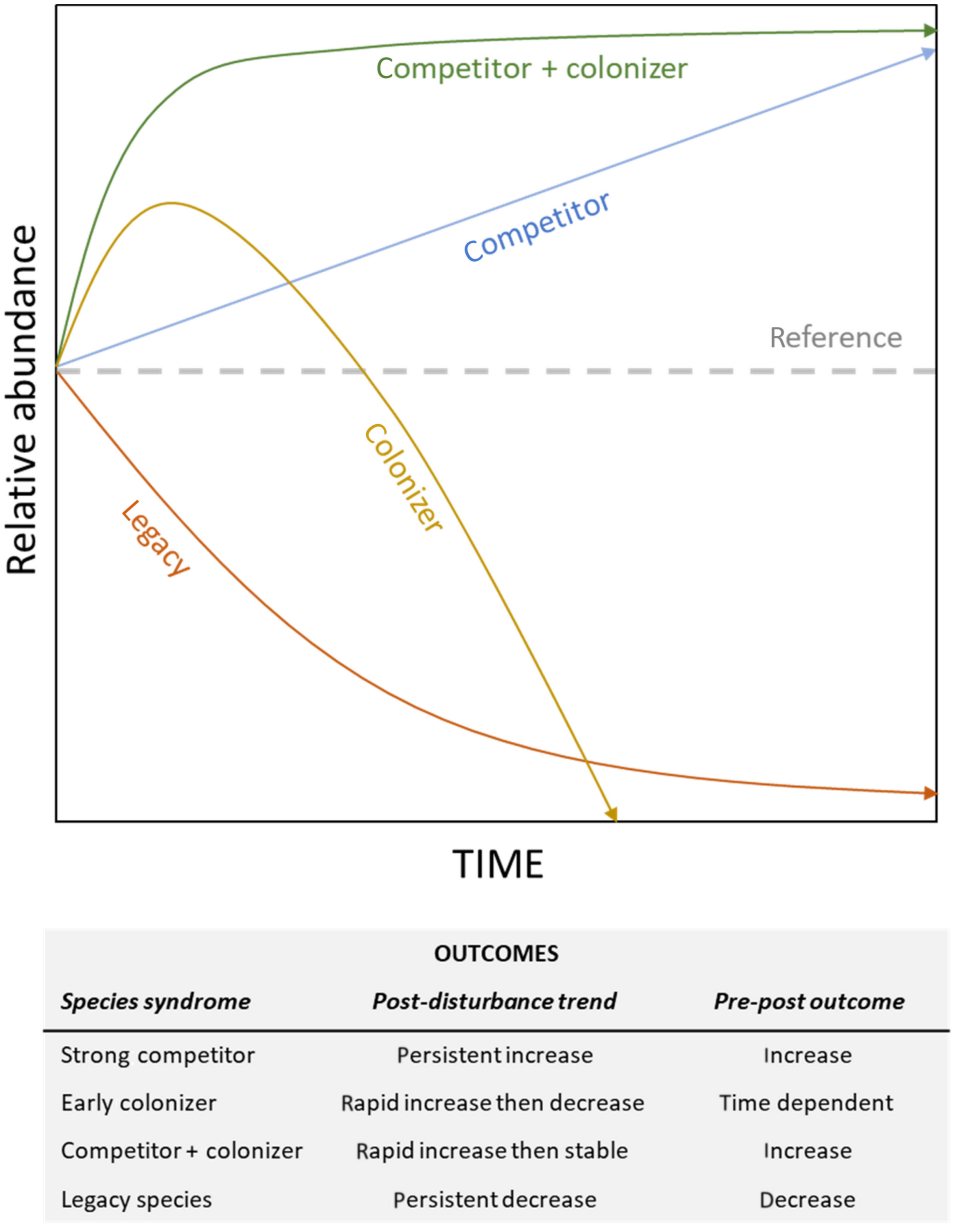
Conceptual diagram illustrating patterns of post‐disturbance community assembly versus a reference line of no change. Both a strong competitor and a legacy species would initially be outcompeted by the early colonizers, but the strong competitor would gradually increase in prevalence through time while the legacy species would continue to decline. The post‐disturbance outcome represents the post‐disturbance change in proportion of plant community within disturbed plots through time (Figures A10, A11), while the pre → post outcome represents the ultimate change from the pre‐disturbance condition to the last post‐disturbance condition (Figures 2, 3, 4). Note that each scenario would have a unique combination of post trend and pre → post outcome.

We explored how native and invasive‐dominated plant communities recovered in the first 21 months following disturbance, and whether species were initially present because they were strong competitors, early colonizers, or both. If species were strong competitors, we expected an initial post‐disturbance shift toward dominance by early colonizing species, followed by a gradual return toward the initial condition (Figure 1). Alternatively, if a species was an early colonizer, we expected community composition to quickly return to pre‐disturbance conditions and remain relatively constant thereafter. Finally, if a species was both a superior competitor and an early colonizer, we expected it to take over disturbed sites, even when other species briefly show up during post‐disturbance succession. Obviously, these mechanisms are not mutually exclusive and likely exist on a continuum (HilleRisLambers et al., 2010); however, insight into these various mechanisms will help predictability in the face of increasing anthropogenic pressures. While we note that this is a relatively short study, making it difficult to address long‐term dynamics, we feel that in this productive ecosystem we were able to ask whether patterns began to emerge. We also focused on obtaining fine‐scale data that could be used to discern patterns—for example, species that are able to recruit into disturbed areas but do not survive past a few months—that can be lost in studies with long intervals between sample dates.

To help elucidate patterns of community assembly following disturbance, we performed a disturbance experiment within six dominant vegetation types and asked: (1) Which patterns of community assembly (Figure 1) do common species exhibit within each of the different vegetation types? (2) Related, how stable were the different community types in the face of disturbance? and (3) Was native regeneration limited after disturbance, and if so, was it limited by dispersal or competition?

## 2 MATERIALS AND METHODS

### 2.1 Study area

All study plots were located within the The Keʻāmuku Maneuver Area (KMA), a 9227‐ha parcel that was added to the Army Pōhakuloa Training Area on the Island of Hawai‘i in 2006. KMA was owned by the Parker Ranch from approximately 1847 but grazing from King Kamehameha's feral cattle may have started earlier (Bergin, 2004). The property was purchased by the military and cattle grazing was phased out in 2009 and since that time, parts of the area have been grazed by feral introduced ungulates such as domestic goats (*Capra aegagrus hircus*), domestic sheep (*Ovis aries*), and mouflon sheep (*O.  gmelini musimon*) (Army Garrison‐Hawaii, 2010). KMA spans a range of elevation (800–1700 m) and thus a range of mean annual temperature (MAT, 12–19°C) and precipitation (MAP, 530–720 mm) (Giambelluca et al., 2013). Underlying substrates are volcanic flows ranging from 14,000 to >250,000 years old. A mix of seasonally dry vegetation, strong winds, and military training makes the area prone to wildfire. KMA is a dry landscape that is prone to stochastic heavy rainfall events. It consists primarily of non‐native, invasive perennial grasslands (Table 1) with some scattered native shrublands (dominated by *Dodonaea viscosa* but also includes native shrubs *Sida fallax* and *Waltheria indica*) and native grassland (dominated by the perennial *Eragrostis atropioides*) areas. The noxious pasture weed *Senecio madagascariensis* can be found throughout the area. Non‐disturbed “reference communities” are not available in this arid landscape, but recent evidence points to later successional communities being a mix of native shrubs and grasses dominated by *D. viscosa* and *E. atropioides* with scattered trees (Kinney et al., 2015).

**TABLE 1:** The six dominant plant species used to define vegetation types for the disturbance experiment.

Species	Common name	% cover	Invasive/native	Functional group/Dispersal traits
*Cenchrus ciliaris*	Buffel grass	6.22	Invasive	C_4_ perennial bunch grass
				Wind‐dispersed seed
*Cenchrus clandestinus*	Kikuyu grass	33.82	Invasive	C_4_ perennial rhizomatous grass
				Disperses vegetatively via runners
*Cenchrus setaceus*	Fountain grass	32.50	Invasive	C_4_ perennial bunch grass
				Wind‐dispersed seed
*Melinis repens*	Natal red top	11.30	Invasive	C_4_ perennial bunch grass
				Wind‐dispersed seed
*Dodonaea viscosa*	ʻAʻaliʻi	3.78	Native	Woody shrub
				Winged, wind‐dispersed seed pods
*Eragrostis atropioides*	Hawaiian lovegrass	1.47	Native	C_4_ perennial bunch grass
				Wind‐dispersed seed

*Note*: Percent cover estimates represent the average cover of each species across 155 random stratified plots established within KMA. All species except *C. clandestinus*, which spreads vegetatively via runners, are prolific seeders. See Figures A1, A2, A3, A4, A5, A6 for photos of seed heads and habit.

We attempted to assess vegetation types using 155 random stratified plots across the landscape and multivariate statistical techniques (Appendix). However, we found that we could not detect species associations in the vegetation sampling data, suggesting that “vegetation types” could simply be described by the dominant grass or shrub species. The ten most abundant species in the percent cover data explained 96% of the vegetative cover across the 155 sampled plots. These species (and % of total plant cover) were *Cenchrus clandestinus* (33.81%), *C. setaceus* (32.49%), *Melinis repens* (11.29%), *C. ciliaris* (6.22%), *D. viscosa* (3.78%), *S. madagascariensis* (2.35%), *Medicago lupulina* (1.96%), *Lepidium africanum* (2.35%), *Eragrostis atropiodes* (1.47%), and *Neonotonia wightii* (1.14%).

### 2.2 Disturbance experiment

**FIGURE A1:**
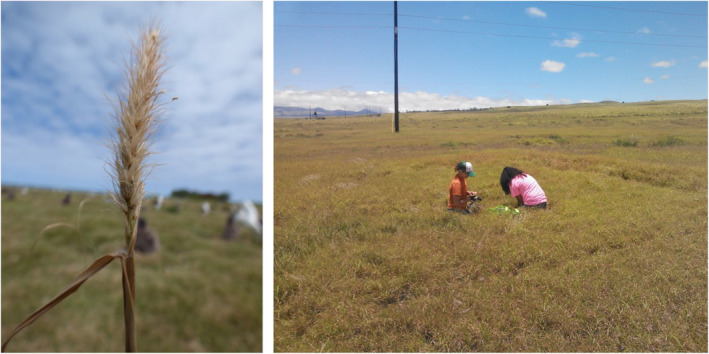
Seed heads (photograph: Forest and Kim Starr) and landscape habit (photograph: S. Yelenik) of *Cenchrus ciliaris* (buffel grass).

**FIGURE A2:**
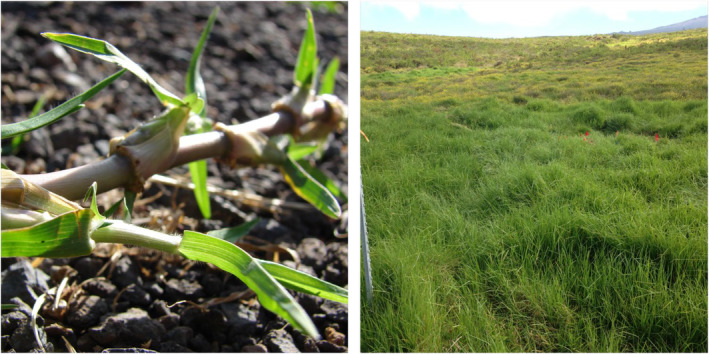
Vegetative runner (photograph: Forest and Kim Starr) and landscape habit (photograph: S. Yelenik) of *Cenchrus clandestinus* (kikuyu grass).

**FIGURE A3:**
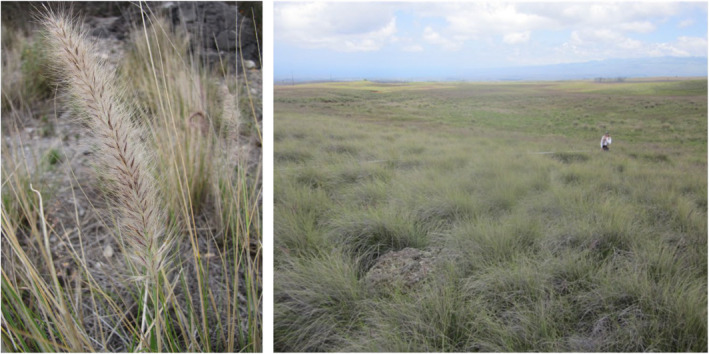
Seed heads (photograph: Forest and Kim Starr) and landscape habit (photograph: S. Yelenik) of *Cenchrus setaceus* (fountain grass).

**FIGURE A4:**
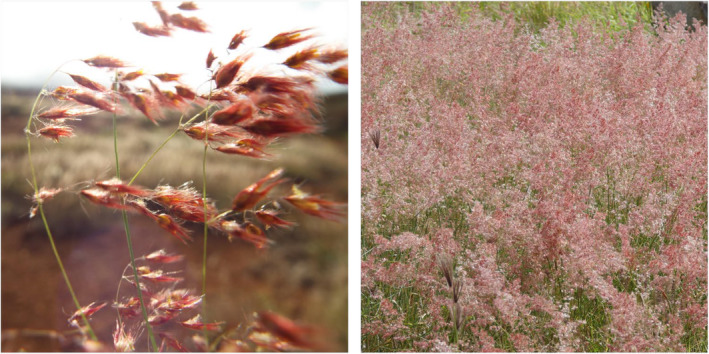
Seed heads and landscape habit (photographs: Forest and Kim Starr) of *Melinis repens* (redtop grass).

**FIGURE A5:**
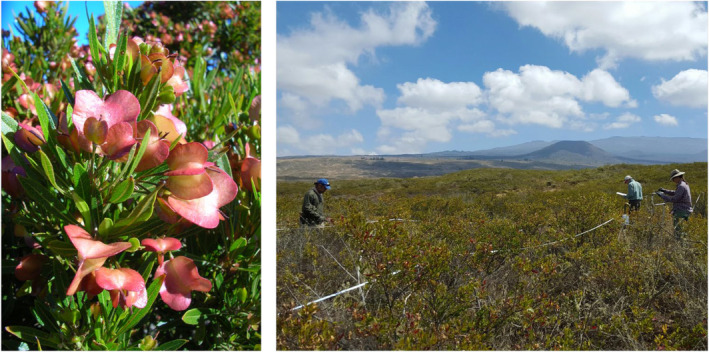
Seed capsules (photograph: Forest and Kim Starr) and landscape habit (photograph: S. Yelenik) of *Dodonaea viscosa* (ʻaʻaliʻi). The beautiful, winged seed pods of this species are commonly used to make lei, signifying some of its cultural importance in Hawaiʻi.

**FIGURE A6:**
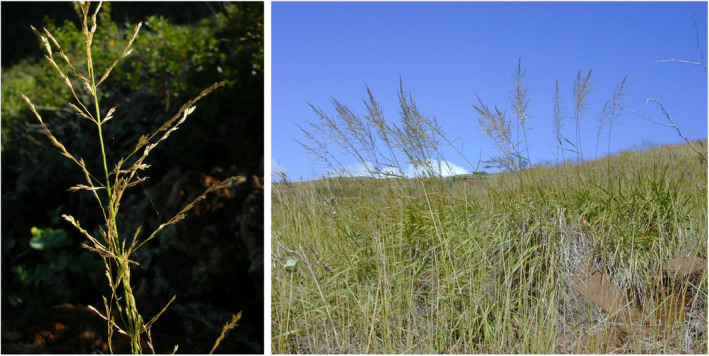
Seed heads and landscape habit (photographs: Forest and Kim Starr) of *Erogrostis atropioides* (Hawaiian lovegrass).

We implemented a disturbance experiment to assess how plant communities would reassemble (see also Yelenik, Rose, Cordell, et al., 2022). We chose the five most dominant species from the random plot survey as the vegetation types, which included four invasive grasses (*C. ciliaris*, *C. clandestinus*, *C. setaceus*, and *M. repens)* and one native shrub (*D. viscosa*). In addition, we included *E. atropioides*, the ninth most dominant species at KMA to include a native species of the same functional group (bunchgrass) as many of the invasive species (Table 1, Figures A1, A2, A3, A4, A5, A6).

**FIGURE A7:**
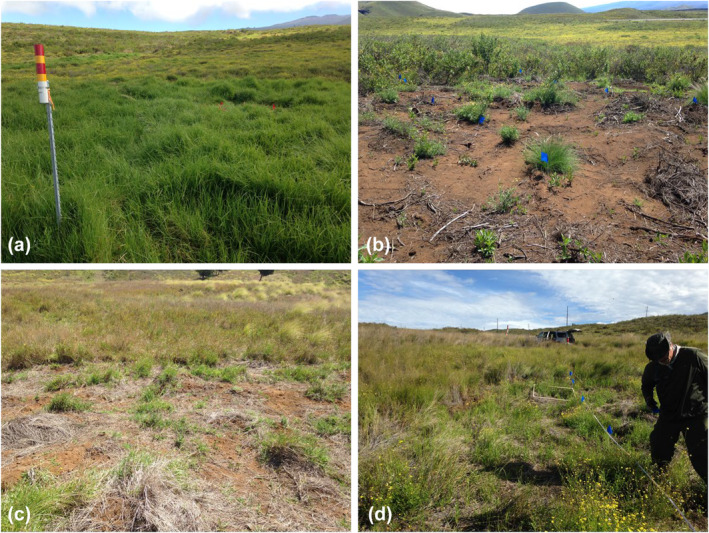
Photographs of examples of experimental disturbance plots for four of the vegetation types. Panels show (a) non‐native, invasive *Cenchrus clandestinus* (kikuyu) grassland, (b) native *Dodonaea viscosa* (ʻaʻaliʻi) shrubland, (c) non‐native, invasive *C. ciliaris* (buffel grass) grassland and (d) native *Erograstis atropioides* (Hawaiian lovegrass) grassland. Photographs were taken between 1.5 and 3 months after the mechanical disturbance event in July 2014, and show the disturbed plot in the foreground, and the undisturbed plot in the background. Note the fast regrowth of *C. clandestinus* grass in the foreground of (a), which was disturbed only 1.5 months prior to the picture. All photographs were taken by S. Yelenik.

**FIGURE A8:**
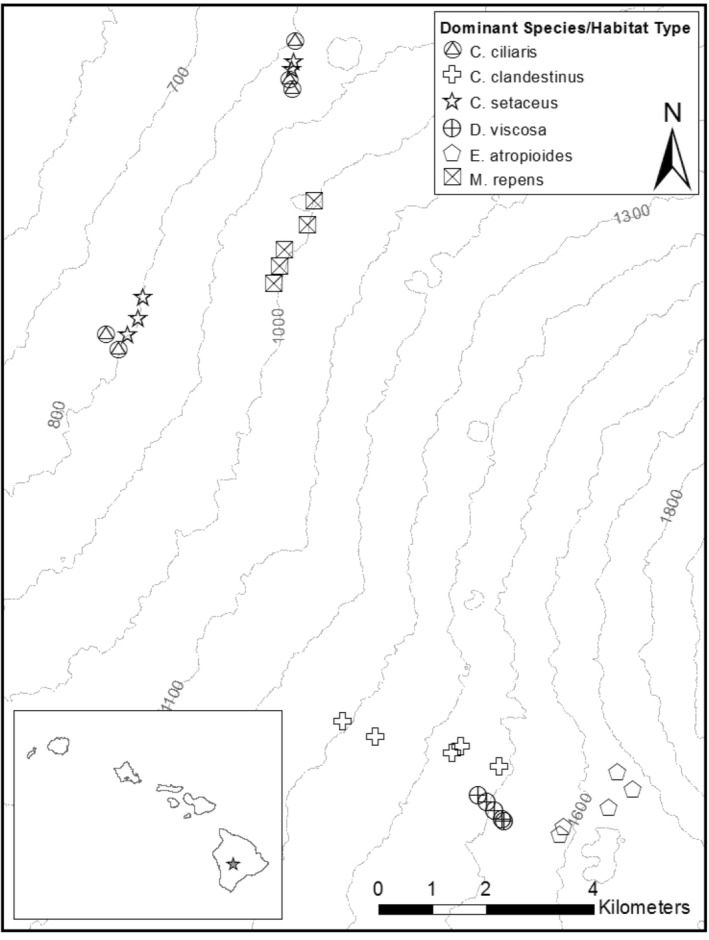
Locations of disturbance experiment plot replicates in the different vegetation types: invasive *Cenchrus ciliaris* (buffel) grassland, invasive *Cenchrus clandestinus* (kikuyu) grassland, invasive *Cenchrus setaceus* (fountain) grassland, native *Dodonaea viscosa* (ʻaʻaliʻi) shrubland, native *Eragrostis atropioides* (Hawaiian lovegrass) grassland, and invasive *Melinis repens* (redtop) grassland.

**FIGURE A9:**
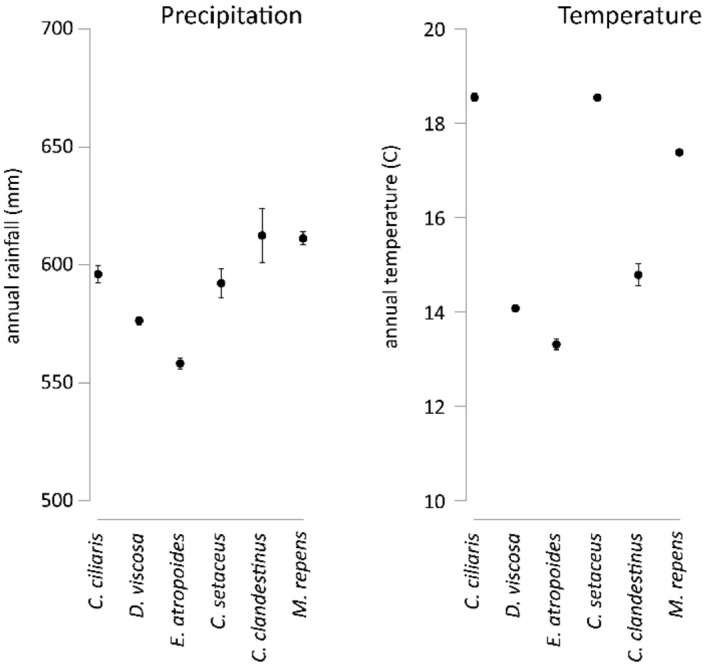
Average precipitation and mean annual temperature in disturbance sites. Averages were taken for data from 1920 to 2012 using the Hawaiʻi Rainfall Atlas (Giambelluca et al. 2013).

We used a split‐plot BACI (before, after, control, and impact) design as a framework for the disturbance experiment (Figure A7). We established five 20‐m × 10‐m plots per vegetation type for a total of 30 plots. Plots were chosen to be a minimum of 100 m apart, on a similar slope and aspect, and in areas that had the greatest percent cover of the target vegetation type. Within each plot, two transects were placed at regular intervals across the 20‐m plot length (at 3 and 7 m across the 10 m width) and percent cover was estimated using a point‐intercept method (Mueller‐Dombois & Ellenberg, 2002; Yelenik, Rose, Cordell, et al., 2022). In July 2014, we randomly selected one 10‐m × 10‐m half of the 20‐m × 10‐m plot to be disturbed (Figure A7). We used large equipment to disturb plots by using the front‐end loader to move vegetation and a fine layer of topsoil (Bobcat®). Debris was left inside the plot. Plots within a vegetation type were spatially aggregated, in part because the vegetation types were dominant on different substrate types and across different climates (Figures A8, A9).

This project was instigated due to interest from the Department of Defense concerning the impacts of military training, including disturbance from large, heavy vehicles and tanks, on erosion and vegetation. While our experiment may not exactly mimic them, it relates to a more broad array of disturbance types that are common in these landscapes, including fire breaks, ranch roads, off‐road vehicles, heavy grazing, and fire. In addition, the replicated, controlled nature of our experiment offers insight that natural experiments often cannot (Diamond, 1986).

We began monitoring vegetation response four months after disturbance (October 2014) and repeated monitoring every 3–4 months. In order to capture finer scale processes (e.g., seedling dynamics) within disturbed areas, we placed five 1 m^2^ quadrats at regular intervals along each of the two initial transects used to collect point‐intercept cover data, for a total of ten quadrats per 10‐m × 10‐m plot. Percent cover of all species within each quadrat was estimated independently by two observers and then averaged. The total cover of all species and substrates summed to 100% within each quadrat. On the final sampling date, we also used the point‐intercept method to quantify percent cover within both disturbed and undisturbed plots. This approach allowed us to relate the two methods (point‐intercept and quadrat) and to compare pre‐disturbance data to post‐disturbance data.

In addition to percent cover, we also quantified plant recruitment. During the first (June 2014, pre‐disturbance) and last (March 2016) sampling visits, we counted the number of native shrubs within every 10‐m × 20‐m plot and assigned each individual to one of four height categories (0–0.5 m, >0.5–1 m, >1–2 m, >2 m). There data were used to calculate stem densities before and after disturbance. To estimate finer scale seedling size class density, we also counted all shrubs and shrub seedlings within each 1 m^2^ quadrat and assigned to one of five height classes (0–5 cm, 5–10 cm, 10–30 cm, 30–100 cm, and >100 cm) during all post‐disturbance quadrat sampling visits.

We were also interested in the fate of native seedlings within each of the dominant vegetation types, where they would likely be dispersal limited and have low natural recruitment, making it impossible to watch natural seedling dynamics. Therefore, we planted five seedlings each of *D. viscosa* and *E. atropioides* in each of our disturbed and undisturbed plots in October 2014. These were watered twice a week for 1 month to ensure transplant success. Percent cover, seedling counts, and outplant survivorship were assessed in November 2014, February, June, and November 2015, and finally in March 2016.

### 2.3 Statistical analyses

#### 2.3.1 Species‐specific patterns of plant community assembly

To identify species‐specific patterns of plant community assembly, we conducted two sets of analyses (post‐disturbance trend and pre‐post outcome), each aimed at different indicators of how the four most common species within each vegetation type recovered from plot disturbance relative to its cohorts (Figure 1).

First, we used generalized additive mixed models (GAMM: Zuur et al., 2009) of post‐disturbance plant cover to look for temporal trends in the proportion of the plant community for each species, hereafter *post‐disturbance trend analysis*. GAMMs are a powerful tool for ecological data because they can accommodate errors that are not normally distributed (Bolker et al., 2009), and they can be used to describe non‐linear changes through time by including fitted smoothed functions (Fewster et al., 2000). We conducted GAMM analyses using quadrat sampling data collected during all post‐disturbance sampling from the disturbed plots (October 2014–March 2016). We calculated the ratio of focal plant cover to total plant cover for each quadrat sample to use as a response variable. As quadrat sampling included 10 replicate samples during each plot visit, we included a nested random effect of quadrats within plots to accommodate this plot/visit‐specific replication. We assumed a gaussian distribution and used an identity link function. Model predictions were restricted to the range of the observed data and residual analyisis did not reveal any notable outliers or evidence of nonconstant error variance. GAMMs were performed using the gamm4 package (Wood, 2006) in R, version 4.2.2 (R Core Team, 2022), and the smoothing parameter, *k*, was fixed at four, one fewer than the number of time points.

Second, we used generalized linear mixed models (GLMM) to see if the proportion of the plant community comprised by each species returned to its pre‐disturbance condition following disturbance (hereafter, *pre‐post outcome analysis*). For these analyses, we calculated the proportion of the plant community (the ratio of focal plant cover to total plant cover) along each of the two transects within the 10‐m × 10‐m disturbed plots for both the pre‐disturbance and post‐disturbance point‐intercept data (June 2014 and March 2016). We included a categorical indicator of pre/post disturbance condition as a fixed effect, treating pre‐disturbance as the reference condition, and included a nested random effect of transect within each plot. As such, parameter estimates indicated the change in community composition within disturbed plots from June 2014 to March 2016. Again, we assumed a Gaussian distribution and used an identity link function, as model predictions were restricted to the range of the observed data and residual analyisis did not reveal any notable outliers or evidence of nonconstant error variance.

The results of these two sets of analyses were used to provide insights into species‐specific community re‐assembly syndromes; strong competitor, early colonizer, a combination of the two, or as a legacy species experiencing persisitent declines. Species were assigned to reassembly syndromes (Figure 1) based on patterns of proportional cover following disturbance (post‐disturbance trend), as well as proportional cover relative to pre‐disturbance conditions (pre‐post outcome). Pre‐post outcomes were assigned based on statistical significance (*p* < .10) and the sign of the parameter estimate, while patterns of post‐disturbance trend were evaluated visually by looking at post‐disturbance changes in the proportion of the plant community through time. For example, a species that exhibits a significant decrease in relative abundance is likely a legacy species or an early colonizer, depending on the pattern of post‐disturbance trend. As a given plant can have different traits that facilitate different community reassembly syndromes through time and under different circumstances, using a visual approach to interpret patterns of post‐disturbance trend, although somewhat subjective, is useful for comparing accross different species and vegetation types. While these classifications may be somewhat subjective, we found this set of rules helpful to help us classify and interpret our results.

### 2.4 Recruitment and survival of native species

To look for patterns of recruitment, we calculated the difference between pre‐disturbance (June 2014) and the last post‐disturbance (March 2016) stem density for *D. viscosa*, *S. fallax*, and *W. indica* within each plot and treatment combination. Only plants less than 1 m tall were included in stem density analyses as it is unlikely any post‐disturbance seedlings would have grown to that height over the duration of the study. Using the pre‐disturbance to post‐disturbance density measures, we developed five general linear models (i.e., null, treatment, vegetation type, additive effects of treatment and vegetation type, and an interaction between treatment and vegetation) for each species to evaluate the possible effects of treatment and vegetation type on changes in stem density. We assessed support for each of these models by calculating relative weights using AICc (corrected Akaike information criterion). We also visually assessed sampling time points to make note of pulses of seedling species in disturbed plots of the different vegetation types (Figure 5).

To analyze survivorship of seedlings outplanted in different dominant vegetation types, we used survivorship analysis (glm with exponential distribution, and a loglink estimation method, JMP®, 2019) . We used AIC to pick the best‐fit distribution and conducted independent analyses for the two outplanted species (*D. viscosa* and *E. atropioides*) using dominant vegetation type and disturbance as model effects.

### 2.5 Overall effects of disturbance on species cover and dominance

To elucidate overall differences in percent cover resulting from disturbance while controlling for background changes found within paired control plots, we again used GLMMs. We calculated the change in percent cover between pre‐disturbance (July 2014) and the last post‐disturbance (March 2016) samples for the four most common species within each vegetation type and for bare ground. This was done for each of the two transects independently within paired control (control_post_–control_pre_) and disturbed (disturbed_post_–disturbed_pre_) plots. We used the resulting change in cover as a response variable within GLMM's, including a fixed effect of treatment and nested random effects of transect within each plot to account for within‐plot variation. We treated control plots as the reference condition, so parameter estimates were indicative of the change in percent cover resulting from disturbance. Similar to previous percent cover analyses, models used a gausian distribution and residuals did not reveal any notable outliers or evidence of nonconstant error variance.

In addition, we also looked for overall changes in community structure in the point‐transect cover data by ordinating on percent cover by species via multidimensional scaling. We used 22 species that made up 75% of the total cover in the pre‐ and posttreatment point‐transect surveys (excluding bare ground). To detect systematic changes in community type with treatment we fit a mixed‐effects model (nesting within vegetation type) with the first two ordination scores as a response. We looked at five models: a null model of random variation within vegetation type, time and treatment each individually, time and treatment together in an additive model, and a time and treatment interaction (which would indicate a treatment effect on community structure). We assessed the evidence for each of these models by calculating relative weights using AICc. To look for differences in the relative *magnitude* of change in community structure, we fit a similar mixed‐effects model to look for treatment effects.

## 3 RESULTS

### 3.1 Species‐specific patterns of plant community assembly

**FIGURE 2:**
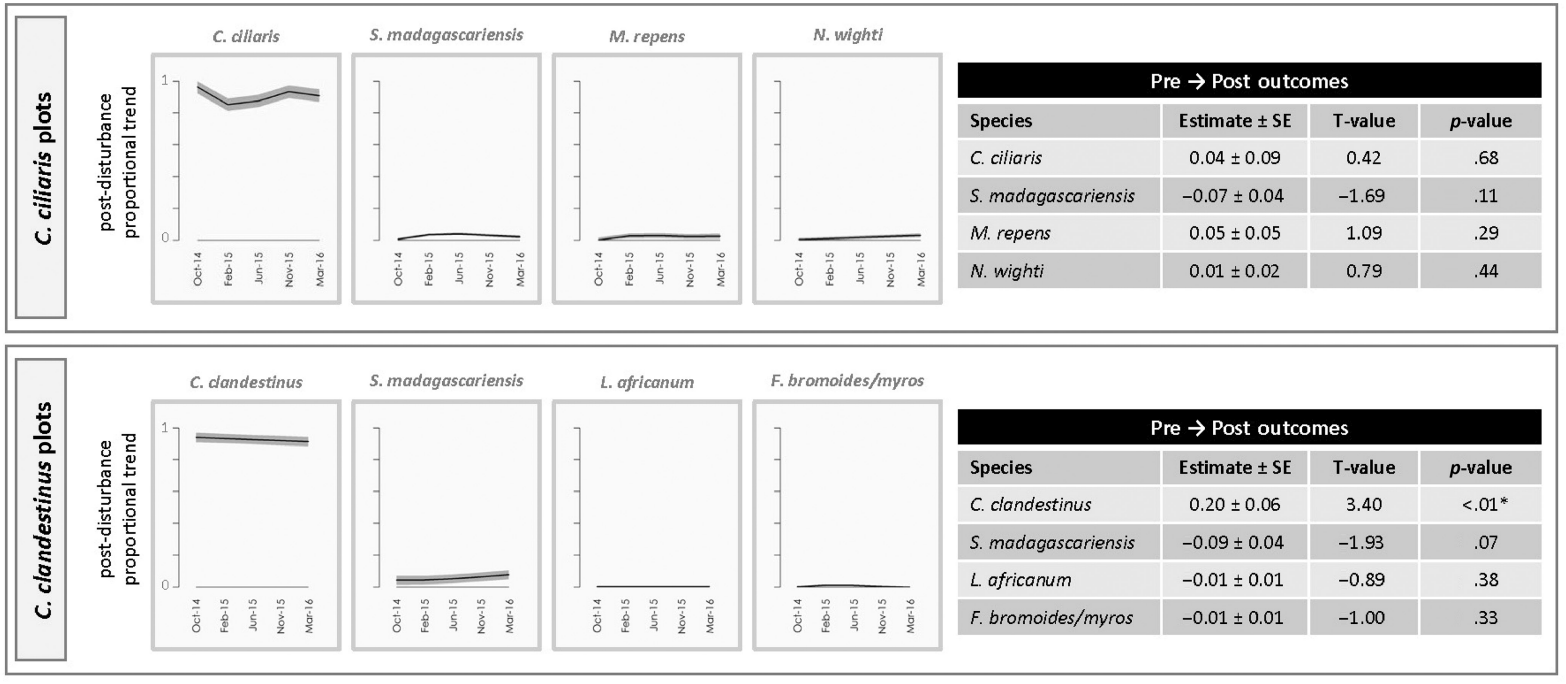
Patterns of community assembly within plots dominated by invasive *Cenchrus ciliaris* and *Cenchrus clandestinus* following disturbance. The figures on the left within each group show post‐disturbance changes in the proportion of total plant cover for each of the four most common species. These figures show the results of generalized additive mixed models for each species (post‐disturbance trend analyses). Black lines indicate smoothed predictions of the proportion of total plant cover comprised by the focal species, while the surrounding gray shading represents a single standard error (SE) around the estimates. The tables present generalized linear mixed model results for relative changes in the proportion of total plant cover comprised by the focal species from prior to disturbance (June 2014) to the last sampling period (March 2016). Parameter estimates reflect the extent to which the proportional contribution of each species changed over time.

**FIGURE 3:**
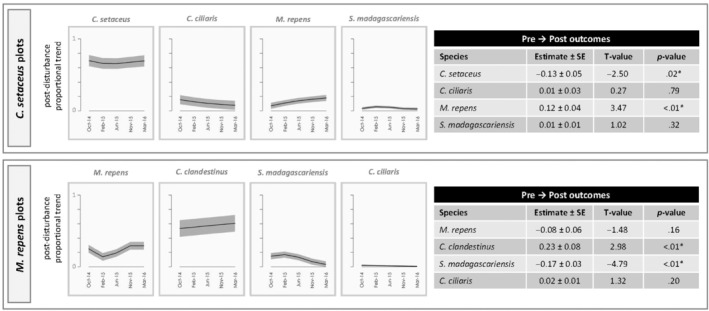
Patterns of community assembly within plots dominated by invasive *Cenchrus setaceus* and *Melinis repens* following disturbance. The figures on the left within each group show post‐disturbance changes in the proportion of total plant cover for each of the four most common species. Black lines indicate smoothed predictions of the proportion of total plant cover comprised by the focal species, and surrounding gray shading represents a single standard error (SE) around the estimates. Tables present generalized linear mixed model results for relative changes in proportion of total plant cover comprised by focal species from prior to disturbance (June 2014) to last sampling period (March 2016). Parameter estimates reflect the extent to which the proportional contribution of each species changed over time.

**FIGURE 4:**
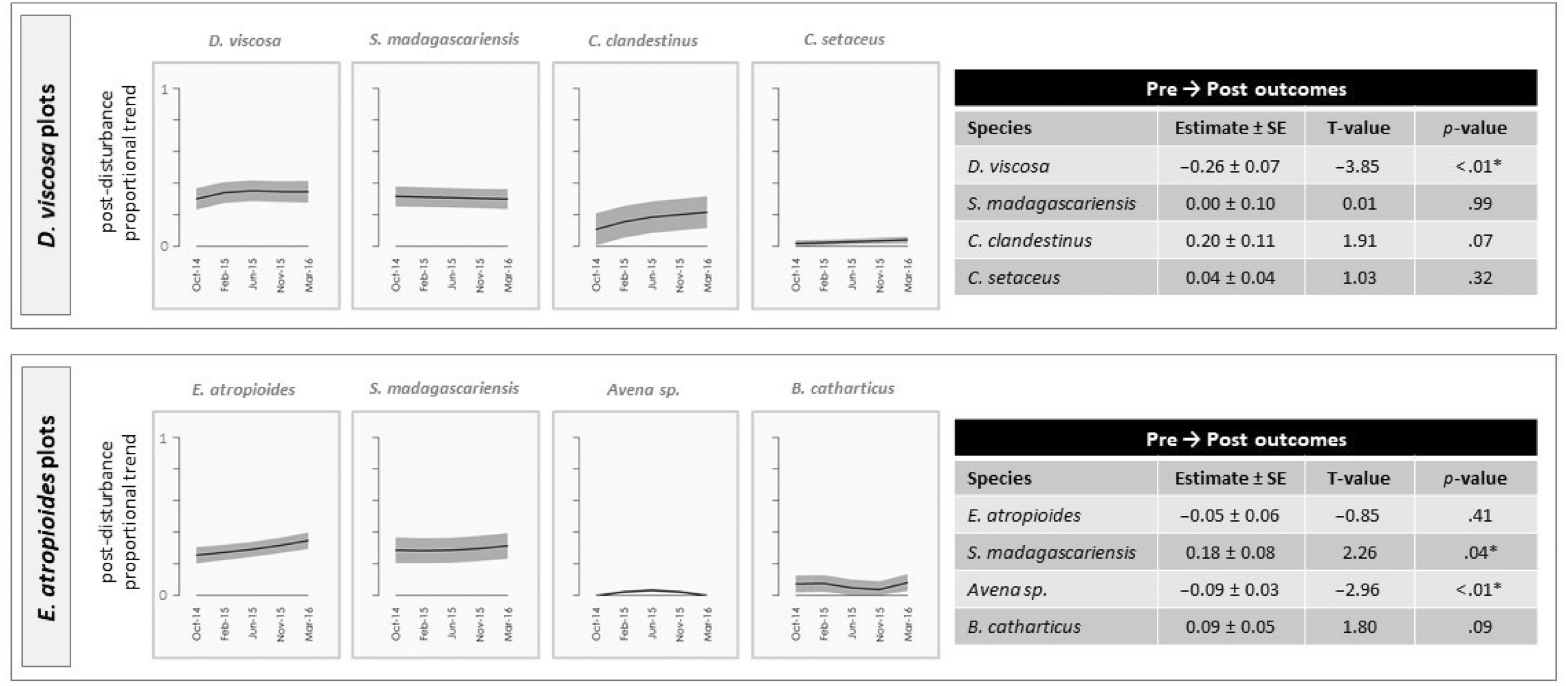
Patterns of community assembly within plots dominated by native *Dodonaea viscosa* and *Eragrostis atropioides* following disturbance. The figures on the left within each group show post‐disturbance changes in the proportion of total plant cover for each of the four most common species. Black lines indicate smoothed predictions of the proportion of total plant cover comprised by the focal species, and surrounding gray shading represents a single standard error (SE) around the estimates. Tables present generalized linear mixed model results for relative changes in proportion of total plant cover comprised by focal species from prior to disturbance (June 2014) to last sampling period (March 2016). Parameter estimates reflect the extent to which the proportional contribution of each species changed over time.

Within the six focal vegetation types, we found evidence of all four recovery modes (Figure 1): strong competitor, early colonizer, strong competitor + early colonizer, and legacy species or comparatively poor competitor (Table 2, Figures 2, 3, 4). Overall, five species (*C. clandestinus*, *C. ciliaris*, *C. setaceus*, *M. repens*, and *S. madagascariensis*) were commonly encountered within multiple vegetation types, and another seven species (*D. viscosa*, *E. atropioides*, *L. africanum*, *F. bromoides*/*myros*, *N. wightii*, *Avena* sp., and *B. catharticus*) were common in no more than one of the vegetation types. Of these 12 common species, three taxa exhibited a single recovery mode each, three taxa showed evidence of multiple recovery modes, and another five taxa were common enough for analysis in one or more vegetation type, but patterns of recovery were inconclusive owing to low proportional cover following disturbance. Although only half of the species showed a single mode of recovery, two of the species that showed evidence of different recovery strategies within different ecological contexts (*C. clandestinus* and *M. repens*) were consistently a combination of early colonizer and/or strong competitor. The most variable species was *S. madagascariensis*, which exhibited signs of three different recovery modes depending on habitat type and community composition. Within *E. atropioides*‐dominated plots, *S. madagascariensis* exhibited recovery patterns consistent with both an early colonizer and a strong competitor, such that the species' proportional contribution to total plant cover quickly returned to pre‐disturbance levels and did not change over time. In contrast however, within *M. repens*‐dominated plots, *S. madagascariensis* exhibited patterns consistent with being a legacy species, including post‐disturbance declines and a significant reduction in the proportion of cover by the end of the study. The *M. repens* sites had greater MATs than the other habitat types (Figure A9).

**TABLE 2:** Recovery modes for common species within each vegetation type.

	*CENCIL*	*CENCLA*	*CENSET*	*DODVIS*	*ERAATR*	*MELREP*	*SENMAD*
Vegetation type							
*Cenchrus ciliaris*	col (1)					inc (3)	inc (2)
*Cenchrus clandestinus*		col (1)					com (2)
*Cenchrus setaceus*	col (2)		inc (1)			com + col (3)	inc (4)
*Melinis repens*	inc (4)	com + col (2)				com (1)	leg (3)
*Dodonaea viscosa*		com + col (3)	inc (4)	com (1)			inc (2)
*Erogrostis atropioides*					com (1)		com + col (2)

*Note*: Recovery modes were either inconclusive (inc), or they were assigned to one of the following four categories presented in Figure 1: strong competitor (com), early colonizer (col), strong competitor + early colonizer (com + con), and poor competitor/legacy species (leg). Recovery modes were assigned to each species based on a combination of the post‐disturbance proportional trend (post trend, Figures 2, 3, 4) and the ultimate change in the proportion of total plant cover (pre → post outcomes, Table 3). Note that some species exhibited different recovery modes within different ecological contexts (i.e., different vegetation types). Results are generally presented for the four most dominant taxa within each vegetation type. Species dominance is presented within parenthesis and was determined by averaging percent cover estimates from the first (June 2014) and last (March 2016) visits. Results were deemed inconclusive if post‐disturbance cover was <5% and/or no significant trends were found.

**FIGURE A10:**
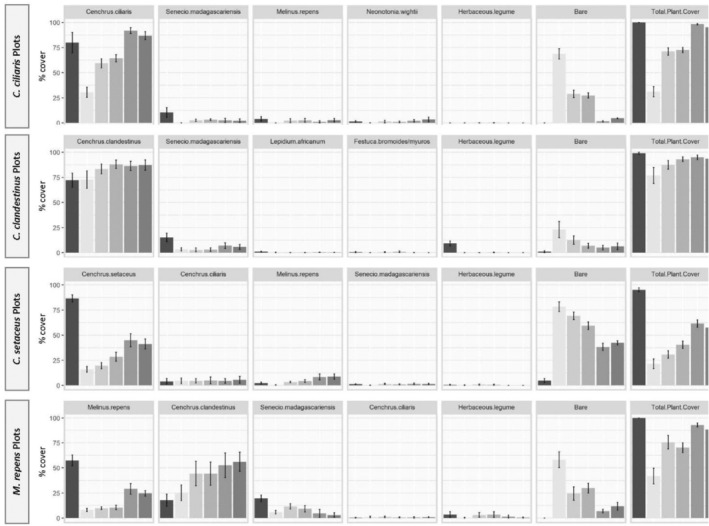
Percent cover before and after disturbance within four invasive‐dominated vegetation types. Percent cover estimates were collected using point‐intercept sampling, while post‐disturbance cover estimates were collected using quadrat sampling methods.

*Melinis repens* also exhibited patterns attributed to two different recovery modes within different plot types. In *M. repens‐*dominated plots, recovery of the dominant species was indicative of a strong competitor, such that it showed increases in its contribution to total plant cover through time following disturbance (Figure A10), and its proportional contribution to overall plant cover returned to pre‐disturbance levels (Figure 3). In contrast, within *C. setaceus*‐dominated plots, *M. repens* exhibited recovery patterns consistent with a competitor + colonizer. Specifically, an increasing post‐disturbance contribution to total vegetation cover combined with an increase in its proportional cover relative to pre‐disturbance levels.

### 3.2 Recruitment and survival of native species

**FIGURE 5:**
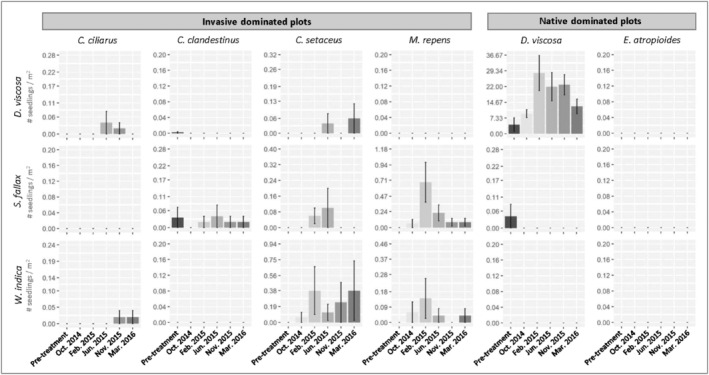
Seedling density prior to, and following, disturbance within KMA sampling plots. *Dodonaea viscosa*, *Sida fallax*, and *Waltheria indica* seedling density were calculated by dividing the total number of seedlings within each plot by the plot area $\left(\frac{\#\text{stems}\leq 1m\;\text{tall}}{\text{plot area}}\right)$. Note that y‐axes vary in scale to improve presentation.

AIC model selection for *D. viscosa* or *S. fallax* change in seedling density (difference in number of seedlings between pre‐disturbance and final post‐disturbance sampling time points) did not find evidence to support an effect of vegetation type, treatment, or a combination of the two on native plant recruitment. In both cases, the top model carried most of the model weight and did not include either covariate (*D. viscosa*; *k* = 1, AICcWt = 0.89, *S. fallax*; *k* = 1, AICcWt = 0.88). In contrast, the top model for *W. indica* included an effect of treatment (*W. indica*; *k* = 3, AICcWt = 0.64) and was followed by a model with both treatment and vegetation type effects (*k* = 7, AICcWt = 0.15). The effect of treatment on *W. indica* was driven by increased stem densities in disturbed relative to control plots within *C. cilliarus* (control: ∆ density = 0.00 ± 0.00, disturbed: ∆ density = 0.02 ± 0.01), *C. setaceus* (control: ∆ density = 0.00 ± 0.00, disturbed: ∆ density = 0.23 ± 0.12), and *M. repens* (control: ∆ density = 0.00 ± 0.00, disturbed: ∆ density = 0.02 ± 0.02) dominated plots. Periodic monitoring (all sampling time points), however, did reveal temporal changes in seedling density following disturbance (Figure 5). For example, there were pulses of *D. viscosa* seedlings in *C. ciliaris* and *D. viscosa* vegetation types, as well as pulses of *S. fallax* seedlings in *C. clandestinus*, *C. cetatceus*, and *M. repens* vegetation types. Despite these pulses in seedling recruitment, there was enough variability among plots and seedling die‐off that analyses did not reflect these changes.

**FIGURE 6:**
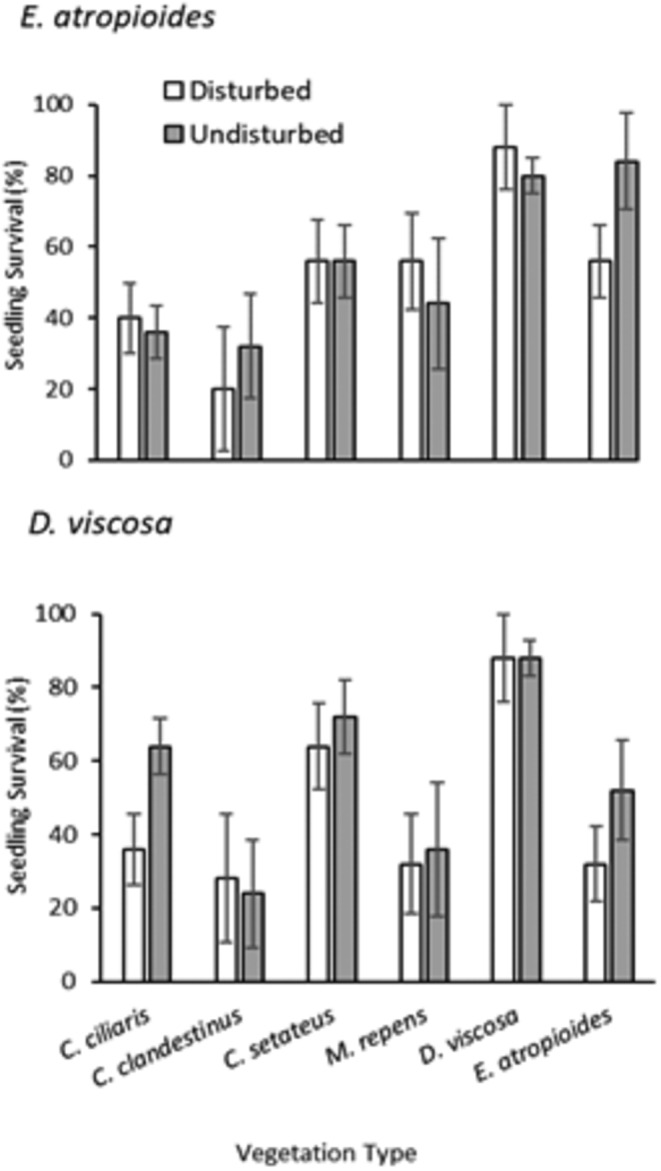
Survival of *Dodonaea viscosa* and *Eragrostis atropioides* seedlings after 16 months in different vegetation types and disturbance regimes. Bars show means ± 1SE (standard error). GLM results: *D. viscosa* seedlings—vegetation type effect: χ^2^ = 46.46, *p* < .001, disturbance effect: χ^2^ = 4.78, *p* = .029; *E. atropioides* seedlings—vegetation type effect: χ^2^ = 24.69, *p* < .001, disturbance effect: χ^2^ = 0.22, *p* = .638.

Survival of outplanted *viscosa* and *E. atropioides* seedlings differed among vegetation types, with *D. viscosa* seedling survival showing an effect of disturbance (Figure 6). Both seedling species showed the lowest survival overall in *C. clandestinus* plots (26% for both seedling species) and the highest overall survivorship in *D. viscosa* plots (88% and 84% for *D. viscosa* and *E. atropioides*, respectively). However, *E. atropioides* achieved high survivorship in its own habitat (70%), while *D. viscosa* seedlings did not survive as well (42%) in this habitat type.

### 3.3 Overall effects of disturbance on species cover and dominance

**FIGURE A11:**
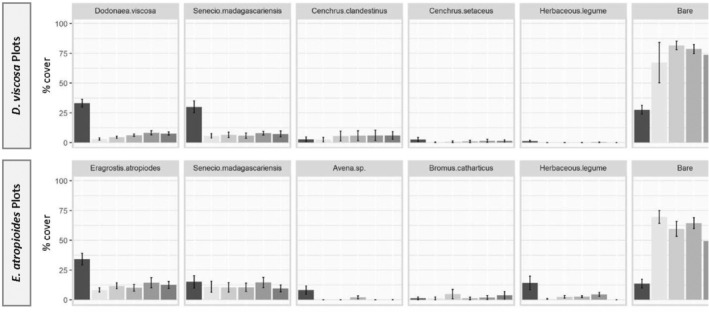
Percent cover before and after disturbance within two native dominated vegetation types. Percent cover estimates were collected using point‐intercept sampling, while post‐disturbance cover estimates were collected using quadrat sampling methods.

By the final sampling effort, disturbance generally increased the amount of bare ground relative to undisturbed control plots, though these patterns were only significant within two vegetation types: *C. setaceus* and *D. viscosa* (Table 3). Within plots dominated by invasive grasses *C. setaceus* and *M. repens*, the native shrub *D. viscosa*, and the native grass *E. atropioides*, disturbance resulted in substantial declines in percent cover of the dominant species (Figures A7, A11). Despite substantial losses in cover within these communities, we did not find evidence for shifts in species dominance resulting directly from disturbance. Although *M. repens* cover increased within *C. setaceus* plots and *B. catharticus* increased within *E. atropioides* plots (Figures A3, A4), these increases were not enough to shift plot dominance (Figures A3, A4). There was an increase in *C. clandestinus* cover within *M. repens* plots, but since this shift happened within both disturbed and undisturbed plots, this pattern was masked within the BACI analysis (Table 3).

**TABLE 3:** BACI analysis results exploring the effects of disturbance on overall percent cover for the four most common species and bare ground (the compliment of total plant cover) within each of the six vegetation types exposed to experimental disturbance.

Vegetation type	Cover type	Estimate ± SE	Test statistic	*p*‐Value
*Cenchrus ciliaris* (Buffel grass)	*Cenchrus ciliaris*	−0.20 ± 6.40	−0.03	.98
	*Senicio madagascariensis*	1.20 ± 3.35	0.36	.73
	*Melinus repens*	2.40 ± 3.38	0.71	.50
	*Neonotonia wightii*	1.40 ± 1.05	1.33	.22
	Bare ground	0.40 ± 0.92	0.43	.67
*Cenchrus clandestinus* (Kikuyu grass)	*Cenchrus clandestinus*	−3.40 ± 4.93	−0.69	.51
	*Senicio madagascariensis*	2.00 ± 4.51	0.44	.67
	*Lepidium africanum*	0.60 ± 0.52	1.15	.28
	*Festuca bromoides/myuros*	−0.20 ± 0.62	−0.32	.75
	Bare ground	1.00 ± 1.49	0.67	.52
*Cenchrus setaceus* (Fountain grass)	*Cenchrus setaceus*	−28.40 ± 6.47	−4.39	<.01*
	*Cenchrus ciliaris*	0.20 ± 4.51	0.04	.97
	*Melinus repens*	11.80 ± 3.08	3.83	<.01*
	*Senicio madagascariensis*	1.40 ± 0.99	1.41	.19
	Bare ground	15.80 ± 2.84	5.56	<.01*
*Melinus repens* (Redtop grass)	*Melinus repens*	−11.20 ± 5.16	−2.17	.05*
	*Cenchrus clandestinus*	4.00 ± 5.58	0.72	.49
	*Senecio madagascariensis*	0.80 ± 3.62	0.22	.83
	*Cenchrus ciliaris*	1.00 ± 1.23	0.81	.43
	Bare ground	2.00 ± 2.21	0.90	.34
*Dodonaea viscosa* (ʻAʻaliʻi)	*Dodonaea viscosa*	−29.40 ± 5.85	−5.03	<.01*
	*Senicio madagascariensis*	−2.40 ± 7.76	−0.31	.76
	*Cenchrus clandestinus*	8.00 ± 4.41	1.81	.10*
	*Cenchrus setaceus*	−0.80 ± 1.96	−0.41	.69
	Bare ground	23.60 ± 6.40	3.69	<.01*
*Eragrostis atropioides* (Hard‐stem Lovegrass)	*Eragrostis atropioides Senicio*	−38.00 ± 4.60	−8.26	<.01*
	*Madagascariensis*	9.40 ± 4.85	1.94	.07*
	*Avena* sp.	0.40 ± 3.51	0.11	.91
	*Bromus catharticus*	8.60 ± 3.42	2.52	.02*
	Bare ground	9.20 ± 5.64	1.63	.13

*Note*: The parameter estimates (Estimates ± SE) indicate generalized linear mixed model predictions of the relative change in percent cover resulting from disturbance. As such, a negative estimate suggests that disturbed plots showed a decrease in cover relative to control plots, and a positive estimate suggests an increase in cover relative to control plots. Estimates were derived from point‐intercept data collected during the first pre‐disturbance visit (July 2014) and the last post‐disturbance visit (March 2016) and fully account for the spatial and temporal associations between treatment and control plots. An asterisk (*) indicates a significant difference between the change in cover over time within disturbed (disturbed_POST_–disturbed_PRE_) and control (control_POST_–control_PRE_) plots.

Although disturbance caused shifts in percent cover for some species, mixed‐effect models of point‐transect cover ordination scores did not provide evidence for overall changes in community composition resulting from disturbance within any of the dominant vegetation types. For the first ordination axis, the best model was a time effect only, with 48% of model weight. The time × disturbance interaction model was marginally worse, with 28% of the model weight. For the second axis, the best model was disturbance only, with 58% of model weight, and the interaction model with only 9%. Comparing the magnitude of community structure changes showed most of the evidence supporting the null model, with only 11% weight for a disturbance effect, suggesting that plant community composition was not appreciably changed due to disturbance. Note that bare ground was not included in the community structure analysis.

## 4 DISCUSSION

Understanding the competitor versus colonizer status of plant species can help predict future dynamics in changing disturbance regimes and help guide management (HilleRisLambers et al., 2010; MacDougall & Turkington, 2005). Here we show that a previously grazed landscape dominated by various invasive species that separate largely due to the environmental filters of substrate age and climate (see Appendix) showed variation in post‐disturbance syndrome (Table 2). This variation in colonizer versus competitor syndrome occurred both between species and also within species among different habitats. This points to the importance of resident communities and their effects on species interactions in shaping a plant community's response to disturbance (Hulvey & Aigner, 2014; Yelenik, Rose, Cordell, et al., 2022). These results are also likely driven by seed availability, or, in the case of *C. clandestinus*, rhizome availability (HilleRisLambers et al., 2010; Hulvey & Aigner, 2014). In other words, showing colonizer tendencies was more likely in plots where a species had been dominant prior to disturbance and thus was not propagule limited (Table 2). At the same time, overall community assemblages, in terms of species dominance, were resilient to disturbance. This suggests that disturbance will likely not change species assemblages on this landscape, including native shrubs. Notably, many native shrubs showed colonizer tendencies, recruiting into invasive‐dominated plots after disturbance, but then were excluded due to competitive interactions. Thus, while both invasive and native species in this system show colonizer tendencies, the invasive grasses may be better competitors for the high resource conditions in the post‐disturbance niche space (Kneitel & Chase, 2004; Pacala & Rees, 1998). We predict that with persistent disturbance, remnant areas of native vegetation may transition to invasive vegetation, but targeted experiments may be able to disentangle these different mechanism (Pacala & Rees, 1998).

### 4.1 A Mosaic of invasive domination with persistent native species

Historical land use and targeted species introductions can select for species assemblages that can tolerate and/or are resilient to high levels of disturbance (HilleRisLambers et al., 2010; Hobbs & Huenneke, 1992), which appears to have happened on this landscape (Leopold & Hess, 2017). Our preliminary vegetation classification (see Appendix) showed that there was a strong effect of substrate, temperature, and rainfall on determining vegetation communities, most of which were dominated by invasive species. The Pacific islands have some of the highest rates of invasive species propagule pressure in the world (Van Kleunen et al., 2015). Between European contact and 1939, the Hawaiian Islands had 183 different species of grasses introduced from different continents and climates (Whitney et al., 1939). Many of these non‐native grasses were introduced to provide high‐productivity forage, and were thus selected for traits that may help with their competitiveness and ability to follow disturbance (D'Antonio & Vitousek, 1992), such as high growth rates, leaf‐area allocation, and high seed production (Van Kleunen et al., 2010). For example, *C. ciliaris* has been distributed globally as a warm‐season pasture grass that could withstand heavy grazing (i.e., disturbance) and drought (Marshall et al., 2012). Prior to becoming a military training facility, KMA was heavily grazed by livestock owned by Parker Ranch. Drastic land‐use change from forest to pasture was common across much of the Hawaiian Islands after foreigners were allowed to buy land titles in 1850, with forest decline reaching a maximum in the late 1800s (Perroy et al., 2016). Put together, the large number of potential non‐native species, high levels of disturbance and chances for spreading, and large climate gradients across Hawaiʻi all likely contributed to the most successful invaders distributing to their best possible niche, which has led to stable pasture assemblages that sort by climate and substrate variables. That these species both showed colonizer and competitor strategies together or in different vegetation types is therefore not that surprising.

The overall resilience of the four invasive vegetation types chosen for the disturbance experiment suggests that many of these species will likely remain dominant, even in areas subject to future disturbance events. Notably, *C. ciliaris* and *C. clandestinus* both returned to pre‐disturbance cover in their respective vegetation types within 1‐year post‐disturbance and exhibited patterns of recovery consistent with an invasive early colonizer (*C. ciliaris*) and an invasive competitor + colonizer (*C. clandestinus*). In fact, *C. clandestinus* not only returned to pre‐disturbance levels within three months of disturbance, but it also increased in proportional cover within disturbed plots over the duration of the study (Figure A1). *Cenchrus clandestinus* is rhizomatous, and because the disturbances were surface level, many of the rooting structures remained intact, potentially leading to rapid resprouting and return of cover (Colman & O'Neill, 1978). *Cenchrus ciliaris* also recovered rapidly but did not reach pre‐disturbance levels until almost 1‐year post‐disturbance. Abiotic variables such as climate and substrate age were primary drivers of vegetation patterns across KMA, and likely affected recovery rates of the different vegetation types (Cox et al., 1988), though we acknowledge that we cannot disentangle species from abiotic effects on regrowth rates given how species correlated with abiotic variables (see Appendix). For example, *C. clandestinus*, which regrew the fastest of any vegetation type, was found in areas that experienced higher precipitation and cooler temperatures than *C. ciliaris* grass (Figure A2), likely because of less soil moisture deficit throughout the year.

Although we found declines in percent cover compared to pre‐disturbance levels for both *C. setaceus* and *M. repens*, these declines were not necessarily indicative of changes in overall species composition within their respective plots. Declines in *C. setaceus* could be attributed to increases in bare ground associated with a slow recovery within that plant community. Similarly, Thaxton et al. (2012) found that *C. setaceus* remained sparse enough in bulldozed dry forest restoration sites for native seedlings to survive and grow. They attributed the negative effects of *C. setaceus* on native woody seedlings to competition primarily for soil moisture, rather than light (Thaxton et al., 2012).

In contrast, while *M. repens* did show some evidence for a shift in dominance within its own vegetation type (Figure A3), this shift was not restricted to disturbed plots in that *C. clandestinus* cover increased during the post‐disturbance period in both disturbed and undisturbed plots. This suggests that a transition in species dominance may have been underway within *M. repens* dominated plant communities irrespective of disturbance. *Melinis repens* was identified as a competitor within its own vegetation type because it showed some evidence for post‐disturbance proportional increases in cover, and it is possible that it will ultimately return to pre‐disturbance levels. However, our results also suggest that *C. clandestinus* may outcompete this species over longer time scales. Notably, pre‐disturbance cover estimates for *M. repens* (57.40 ± 5.45%) were low compared to cover of the other three invasive grasses within their respective vegetation types (*C. ciliaris* = 80.00 ± 10.13%, *C. clandestinus* = 72.20 ± 7.09%, and *C. setaceus* = 86.60 ± 3.60%), suggesting that *M. repens* is not as aggressive as other invasive grasses in this landscape. Similar patterns have been seen elsewhere in Hawaiʻi (de Xavier & D'Antonio, 2017).

Within their own vegetation types, the two native species had the lowest percent cover of any species considered herein, with *D. viscosa* achieving 34% and *E. atropioides* achieving 33% cover in pre‐disturbance plots. Similarly, both species had slow rates of regrowth after disturbance, and never returned to pre‐disturbance cover during this study. Slow growth of native woody species relative to the growth rates of invasive grasses has been shown in multiple studies in Hawaiʻi (e.g., D'Antonio et al., 1998; Questad et al., 2012; Thaxton et al., 2012 and Yelenik et al., 2015). As such, disturbance within these vegetation types may promote bare soil and subsequent erosion, resulting from loss of water infiltration and an increase in overland flow (Ludwig et al., 2005; Perkins et al., 2014). With the possible exception of *M. repens*, *D. viscosa*, and *E. atropioides* appear to be less resilient to mechanical disturbance than the invasive species.

*The natives D. viscosa* and *E. atropioides* were both identified as competitors within their respective vegetation types since their proportional contributions to total plant cover continued to trend upward through 20 months post‐disturbance, suggesting that they may return to pre‐disturbance levels given enough time. For *D. viscosa*, overall decline in cover relative to control plots was partially driven by an increase in bare ground (23.60 ± 6.40%); however, *C. clandestinus* also showed modest increases (8.00 ± 4.41%) in cover within disturbed relative to undisturbed *D. viscosa* plots. As such, *C. clandestinus* may represent a threat to continued dominance by *D. viscosa* in the face of future disturbance, even though the outplanting experiment showed that *D. viscosa* seedlings in their own habitat type. Previous work in Hawaiian dry forests has shown that sites that are favorable to native species also tend to favor an abundance of invasive grasses (Questad et al., 2012), but due to the lower growth rates of native species, invasive grasses remain persistent or take over sites over time (Yelenik, Rose, Cordell, et al., 2022).

Native grass *E. atropioides* also showed significant declines within disturbed relative to control plots, with some increases in bare ground and *B. catharticus*, suggesting a slight shift in species composition. The decline of native grass species in the face of so many introduced grasses was observed as early as 1939 (Whitney et al., 1939); thus, the persistence of *E. atropioides* on a previously grazed landscape suggests that it may be able to maintain some level of abundance in the face of some disturbance over longer time scales (Blackmore & Vitousek, 2006; Leopold & Hess, 2017). Again, the outplanting experiment showed that *E. atropioides* survival was best in native‐dominated habitat types. Yet, the net outcome of these interactions will likely still rely on the relative performance of the native species in the face of invaders in such favorable sites (HilleRisLambers et al., 2010; Questad et al., 2012; Yelenik, Rose, Cordell, et al., 2022).

### 4.2 Seed limitation and colonization

Most of the grasses we focused on are wind dispersed and very fecund, thus prone to long‐distance dispersal, the notable exception being *C. clandestinus* (Holm et al., 1977). As a result of these traits the seed bank within KMA is likely well represented by most of these C_4_ grasses. Although we did not track grass seedling emergence for each of these species, it is probable that given their wind dispersed nature, they were able to disperse to, and then germinate in, open sites across all six vegetation types considered herein. Indeed, buffel grass (*C. ciliaris*) was selected as a forage species in part due to its high seeding rate and ease of propagation (Cox et al., 1988; Stevens & Falk, 2009). A study looking at factors affecting seedling recruitment of *C. setaceus*, including seed limitation and abiotic variables, concluded that drought and soil moisture were more limiting to recruitment than seeds (Goergen & Daehler, 2002).

Our results may have been partly influenced by small plot sizes leading to continued large, post‐disturbance influxes of seeds, particularly of early colonizing species with high seed set or rapid clonal spread (Kotanen, 1997). A dry forest restoration study in Hawaiʻi cleared *C. setaceus*‐ dominated plots that were >2 ha in size (an order of magnitude larger than our disturbed plots) and noted that *C. setaceus* and other invasive species recolonized at very low rates, although we note their sites were drier (Thaxton et al., 2010). Importantly, they noted that seed rain rates of *C. setaceus* remained lower inside than outside of cleared areas. Overall, then, small disturbance sizes may have led to more species being considered colonizers in this study than they would be in larger disturbed/managed areas.

### 4.3 Seedling recruitment and priority effects

While there were post‐disturbance flushes of native shrub seedlings in all dominant vegetation types except *E. atropioides* (Figure 5), most of these seedlings did not survive through 20 months, probably due to fast regrowth of, and competition with, invasive grasses. This highlights that seed of these native shrub species are available; however, it is difficult to ascertain the relative roles of seed‐limitation versus competition without explicit seed addition, grass removal experiments (Pacala & Rees, 1998). The two vegetation types where native seedlings persisted at a density greater than 0.2 seedlings m^−2^ were *C. setaceus* (*Waltheria indica* seedlings: 0.3 seedlings m^−2^) and *D. viscosa* (*D. viscosa* seedlings: 12.6 seedlings m^−2^). These two dominant vegetation types retained high cover of bare ground at of the end of the experiment, and in the case of *D. viscosa*, had either an established seedbank or a local seed source nearby, which probably contributed to comparatively high seedling densities.

It is possible that without a local seed source, recruitment of native shrubs might not have taken place, highlighting the importance of remnant populations and specific starting conditions in post‐disturbance dynamics (Cadotte, 2007; Denslow, 1980; Mordecai et al., 2016). Examples of remnant trees driving post‐disturbance dynamics are fairly common in forested systems (Turner, 2010, e.g., Seidl et al., 2014), but less well studied in the triopics. In Hawaiʻi, studies that used herbicide and weedwhacking treatments at Nakula Natural Area Reserve on Maui found that *D. viscosa* seedlings germinated readily in the short period when invasive grasses had not grown back yet, but none were found in full grass areas or after grasses achieved high cover (Tseng et al., 2021; Warren et al., 2019). In both studies, adult *D. viscosa* plants were nearby to serve as a seed source. Similarly, Goergen and Daehler (2002) found *W. indica* seedlings germinate in seedbank cores taken in *C. setaceus*‐dominated sites and noted that the native shrub made up 5% of the plant cover.

Past work in Hawaiʻi, across various ecosystems and climates, has clearly shown that a major bottleneck to native plant recruitment is competition from invasive grasses (Cabin et al., 2002; D'Antonio et al., 1998; Denslow et al., 2006; Yelenik, Rose, Cordell, et al., 2022; Yelenik, Rose, & Paxton, 2022). *Dodonaea viscosa* has early successional life history traits, including high seed output, and wind‐dispersed seed pods (Harrington & Gadek, 2009). However, it also does poorly with competition from invasive grasses (Yelenik et al., 2015; Yelenik, Rose, Cordell, et al., 2022), and thus it shows a classic competition‐colonization tradeoff. In our study, *D. viscosa* recovered best where it was already dominant, with the highest seedling densities, the highest survival of natural recruits, and the highest growth and survivorship of outplants in *D. viscosa* plots. The fact that outplants showed the highest growth and survival within these plots, suggests comparatively high recruitment was not just a product of having the most seed rain, but that other factors were at play. It is possible that environmental characteristics, species composition, resource availability, or other factors that we did not characterize facilitated *D. viscosa* recruitment within these areas.

Overall, we hypothesize that the native‐dominated communities in our study system that experience a one‐time disturbance will be resilient to change; however, repeated disturbances may lead to type shifts to invader‐dominated communities due to competition from grasses and increasing loss of seed banks and adult seed inputs. Evidence for this includes the pattern that naturally recruiting native seedlings did not survive in invader‐dominated plots. In our study, as has been shown before in Hawaiʻi, native species grew back more slowly than invasive grasses due to lower growth rates and/or competitive interactions (D'Antonio et al., 1998; Warren et al., 2019; Yelenik et al., 2015). Unless mitigated by other factors, this could give grasses time to invade disturbed areas and preempt resources before native species establish (Davis et al., 2000; Pacala & Rees, 1998), ultimately leading to increased invasive grass on the landscape over repeated disturbances. Once invasives become dominant, priority effects, including early colonizing ability, may make it difficult for native woody species to reestablish in the same area (D'Antonio & Vitousek, 1992; Yelenik, Rose, & Paxton, 2022). Repeated disturbances, especially at return intervals that do not allow shrubs to reach reproductive maturity, will lead to diminished seed availability over time, exacerbating competitive effects (Davies et al., 2012; Denslow et al., 2006). Other arid shrublands, such as the U.S. Southwest arid lands, Great Basin sagebrush steppe, and Mediterranean shrublands in Spain, have shown similar patterns of increased degradation with repeated disturbance (Davies et al., 2012; Grigulis et al., 2005; Wilder et al., 2021).

### 4.4 Conclusions

Regardless of colonization/competitor syndrome, the dominant vegetation types that we disturbed were relatively resilient to change. Especially in the case of the invaded communities, this suggests that type conversion from historic conditions represents a type‐shift (D'Antonio & Vitousek, 1992; Suding & Hobbs, 2009; Yelenik, Rose, & Paxton, 2022). This is common in tropical and subtropical old pastures worldwide that were converted from dry and mesic forests for agricultural purposes, especially if they have been in the invader‐dominated pasture state for long periods of time (Griscom & Ashton, 2011; Holl, 1999). While the disturbances that we imposed differ significantly from fire, wildfire resulting from invasive grasses are increasing in Hawaiʻi (Trauernicht et al., 2015) and as such warrant attention in relation to this study. In fact, the 42,000‐acre 2021 Mana Road Fire, the largest fire in Hawaiʻi to date, burned over some of our disturbance sites (Dennison, 2021). Past research in Hawaiʻi shows that fire in invaded grasslands generally results in the return of the same grass species across various ecosystems including those dominated by invasive grasses such as *Melinis minutiflora* (molasses grass), *Schizachyrium condensatum* (tufted beardgrass), and *Megathyrsus maximus* (Guinea grass), as well as one of our study species, *C. clandestinus* (kikuyu grass) (Ainsworth & Kauffman, 2010; D'Antonio et al., 2011; Ellsworth et al., 2014; Hamilton et al., 2021; Trauernicht et al., 2018). Our work suggests that these post‐fire studies may extend to different grass species, and further demonstrates that in the absence of management actions, such grasslands will continue to persist after disturbance, posing continued fire risk (D'Antonio & Vitousek, 1992; Trauernicht et al., 2015). In addition, this pattern of ecosystem stability to disturbance is similar to that of type‐converted rangelands in temperate regions, such as the Great Basin of the continental United States. Here, a combination of invasion and fire have altered landscapes that were dominated by shrubs and perennial grasses into invaded annual grasslands dominated by cheatgrass (*Bromus tectorum*) that are resilient to change (e.g., Chambers et al., 2014). If the desired management goal is native‐dominated ecosystems, such stable states will likely take large inputs of time and resources to alter (Suding & Hobbs, 2009).

## AUTHOR CONTRIBUTIONS

**Stephanie Yelenik:** Conceptualization (lead); data curation (lead); formal analysis (supporting); methodology (lead); project administration (lead); resources (lead); supervision (lead); writing – original draft (lead); writing – review and editing (lead). **Susan Cordell:** Conceptualization (supporting); investigation (supporting); methodology (supporting); project administration (supporting); resources (equal); supervision (equal); writing – review and editing (supporting). **Eli Rose:** Conceptualization (supporting); data curation (supporting); formal analysis (lead); writing – original draft (equal); writing – review and editing (equal).

## Data Availability

Data can be found in Yelenik and Rose (2022).

## APPENDIX A

### A.1 Identifying dominant vegetation types

We sampled 155 random‐stratified 20 m × 20 m plots to determine dominant vegetation types. Random points were generated using ArcGIS 10 and stratified based on geology (Sherrod et al., 2007) and historical use: (1) lava flows less than 65,000 years old (*n* = 44); (2) lava flows over 65,000 years old (*n* = 46); (3) perimeter plots that may have edge effects due to roadways (*n* = 16); (4) pu'us (volcanically derived hills, *n* = 10); (5) slopes greater than 30 degrees (*n* = 11); and (6) Waiki‘i buffer (an area of high intensity grazing that had historically been planted with kikuyu grass, *n* = 27). The number of plots per substrate/land‐use type were chosen using power analysis and are proportionate to the area KMA has classified as this type.

All 155 plots were sampled using a point‐intercept method along three randomly located 20 m north–south transects per plot. A point‐frame was used to drop pins every 20 cm and the first plant species or substrate (soil, litter, etc.) encountered was recorded. Percent cover was calculated per species/substrate, per plot, by dividing total occurrence of each species by the maximum possible pin interceptions. We lumped substrate categories into “bare ground” for analyses. We also counted woody plants in every plot, separating them into species and height classes (0–0.5 m, >0.5–1 m, >1–2 m, >2 m).

We used multivariate statistics to ask whether there were distinct vegetation types (species that associate with each other more than would be predicted by chance). We attempted to reduce the dimensions of the vegetation cover data using multidimensional scaling with a Bray–Curtis distance metric. All methods were attempted using all measured vegetation variables, percent cover measurements only, number of shrubs by species only, and a subset of the percent cover of the top 22 most common species (which composed 75% of the total cover in the dataset).

We used geologic variables describing the substrate age and flow type (Sherrod et al., 2007), and soil carbon content and available water storage (0–30 cm) (Soil Survey Staff, 2013). To describe rainfall and temperature regime we included average monthly precipitation for each month, mean annual precipitation, mean annual temperature, as well as mean minimum temperature in February and mean maximum temperature in August (Giambelluca et al., 2013). These abiotic variables for each station were reduced with a principal component analysis, with the single top component accounting for 46% of variation in the source variables and 90% in the first six variables. To test for spatial autocorrelation, we looked at the distances among stations in vegetation space (using the multidimensional scaling coordinates), the geologic principal components analysis, and the geographic distances between survey stations.

We found evidence for spatial autocorrelation within the data, although the effects of abiotic variables outweighed this. There were statistically significant correlations among all three (substrate age, geographic distance, and vegetation cover, *p* < .0001), demonstrating spatial autocorrelation in both vegetation cover and substrate type. The strongest correlation with *R* ^2^ = .83 was between geographic distance and abiotic variables, with *R* ^2^ values of .42 and .32 between vegetation cover and abiotic distance and geographic distance, respectively. The stronger correlation between the vegetation community composition (measured by the principal component first axis) and abiotic variables than between vegetation cover and geographic distance is evidence that there is an effect of substrate, temperature and rainfall on the vegetation community. There is still significant spatial autocorrelation, however.

Despite these relationships, we did not detect species associations in the vegetation sampling data, suggesting that “vegetation types” could simply be described by the dominant grass or shrub species. De‐centered correspondence analysis of all cover variables and principal coordinate analysis of the common subset of cover variables reduced the variability in the dataset to 17 and 14 dimensions respectively accounting for >90% of the variation in the source variables. Neither method demonstrated clear clusters of species associations, but both indicated communities driven by grass type, or grass type plus *Dodonaea viscosa* shrub cover, with the ten most abundant species in the percent cover data explaining 96% of the vegetative cover across all of the 155 sampled plots. These species (and % of total plant cover) were *Cenchrus clandestinus* (33.81%), *Cenchrus setaceus* (32.49%), *Melinis repens* (11.29%), *Cenchrus ciliaris* (6.22%), *Dodonaea viscosa* (3.78%), *Senecio madagascariensis* (2.35%), *Medicago lupulina* (1.96%), *Lepidium africanum* (2.35%), *Eragrostis atropioides* (1.47%), and *Neonotonia wightii* (1.14%).
